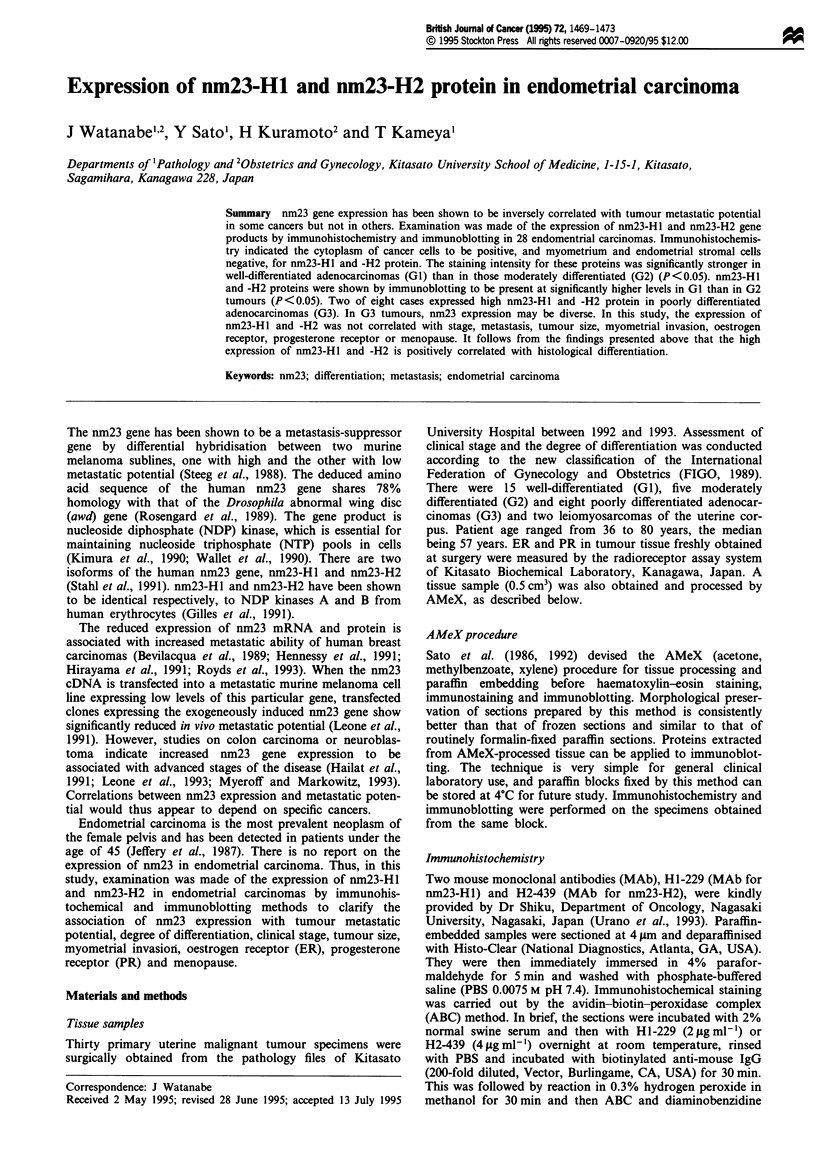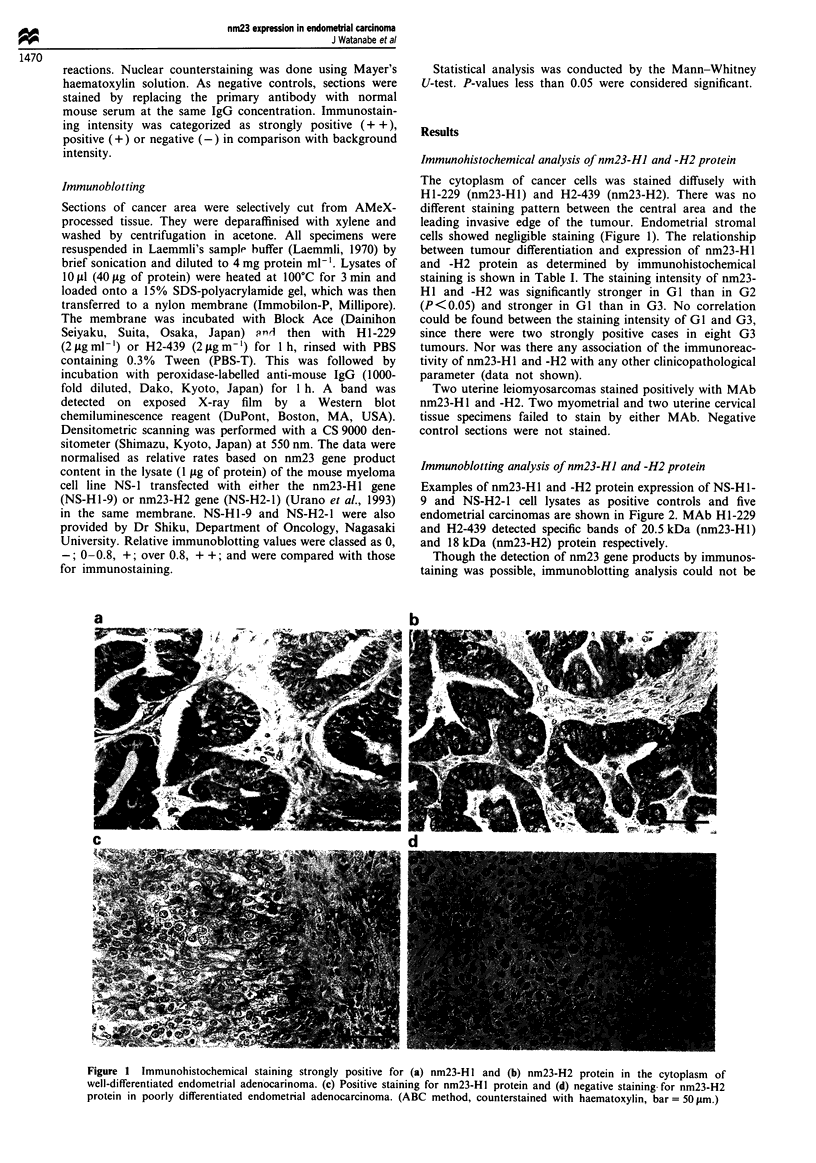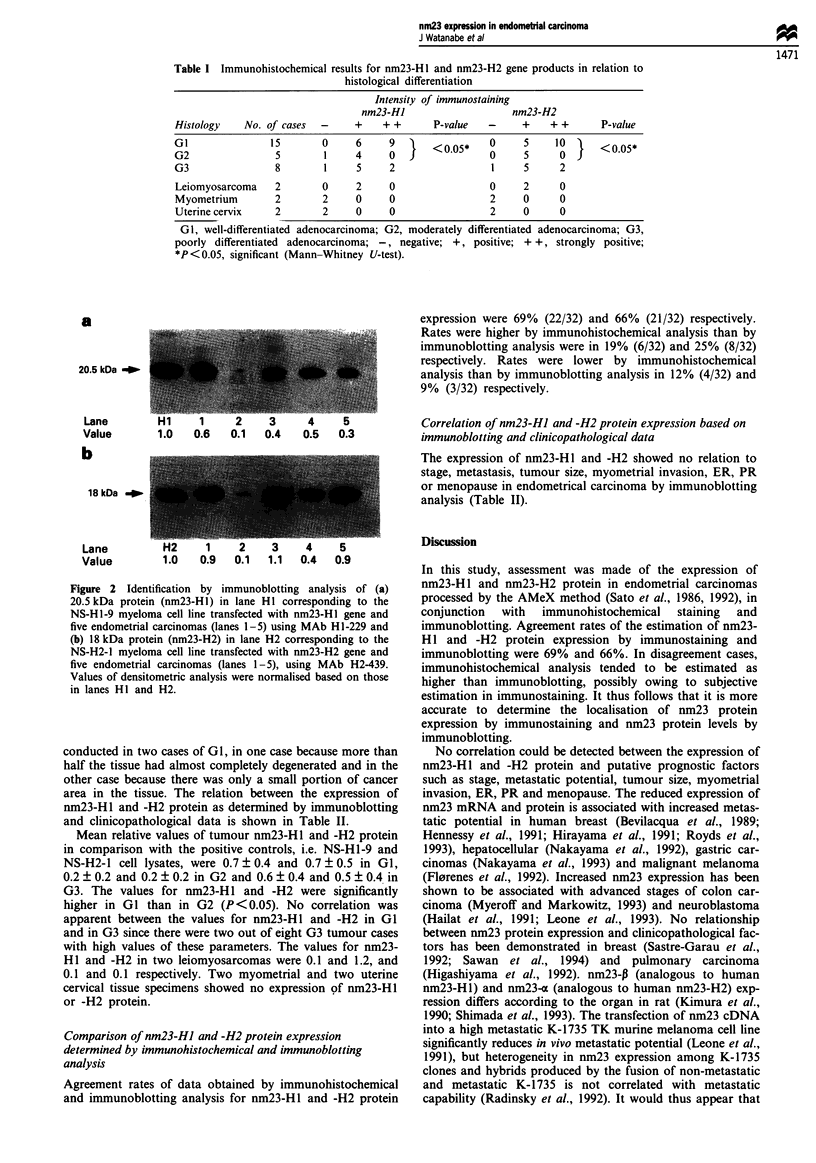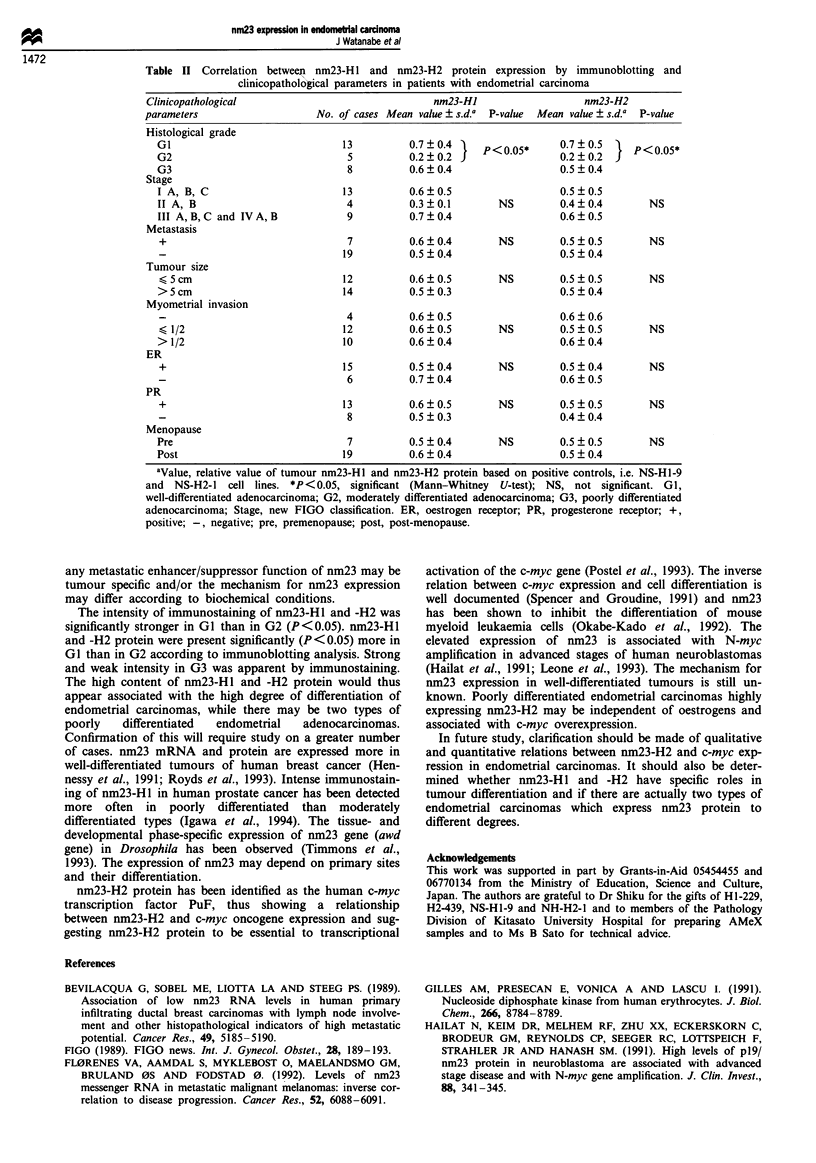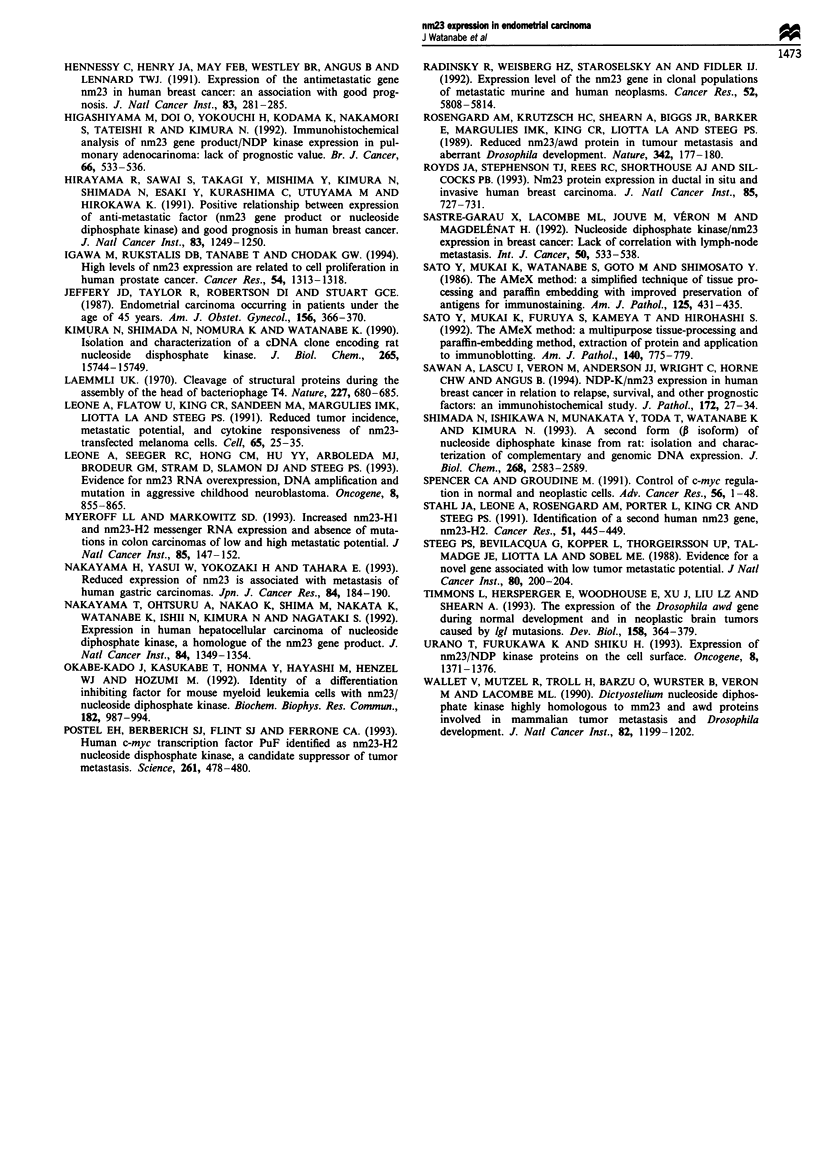# Expression of nm23-H1 and nm23-H2 protein in endometrial carcinoma.

**DOI:** 10.1038/bjc.1995.531

**Published:** 1995-12

**Authors:** J. Watanabe, Y. Sato, H. Kuramoto, T. Kameya

**Affiliations:** Department of Pathology, Kitasato University School of Medicine, Kanagawa, Japan.

## Abstract

**Images:**


British Journal of Cancer (1995) 72, 1469-1473
© 1995 Stockton Press All rights reserved 0007-0920/95 $12.00

### Expression of nm23-H1 and nm23-H2 protein in endometrial carcinoma

J Watanabe[1,2], Y Sato[1], H Kuramoto[2] and T Kameya[1]

*Departments of [1]Pathology and [2]Obstetrics and Gynecology, Kitasato University School of Medicine, 1-15-1, Kitasato, Sagamihara, Kanagawa 228, Japan*

**Summary** nm23 gene expression has been shown to be inversely correlated with tumour metastatic potential in some cancers but not in others. Examination was made of the expression of nm23-H1 and nm23-H2 gene products by immunohistochemistry and immunoblotting in 28 endomentrial carcinomas. Immunohistochemistry indicated the cytoplasm of cancer cells to be positive, and myometrium and endometrial stromal cells negative, for nm23-H1 and -H2 protein. The staining intensity for these proteins was significantly stronger in well-differentiated adenocarcinomas (G1) than in those moderately differentiated (G2) ($P < 0.05$). nm23-H1 and -H2 proteins were shown by immunoblotting to be present at significantly higher levels in G1 than in G2 tumours ($P < 0.05$). Two of eight cases expressed high nm23-H1 and -H2 protein in poorly differentiated adenocarcinomas (G3). In G3 tumours, nm23 expression may be diverse. In this study, the expression of nm23-H1 and -H2 was not correlated with stage, metastasis, tumour size, myometrial invasion, oestrogen receptor, progesterone receptor or menopause. It follows from the findings presented above that the high expression of nm23-H1 and -H2 is positively correlated with histological differentiation.

**Keywords:** nm23; differentiation; metastasis; endometrial carcinoma

The nm23 gene has been shown to be a metastasis-suppressor gene by differential hybridisation between two murine melanoma sublines, one with high and the other with low metastatic potential (Steeg *et al.*, 1988). The deduced amino acid sequence of the human nm23 gene shares 78% homology with that of the *Drosophila* abnormal wing disc (*awd*) gene (Rosengard *et al.*, 1989). The gene product is nucleoside diphosphate (NDP) kinase, which is essential for maintaining nucleoside triphosphate (NTP) pools in cells (Kimura *et al.*, 1990; Wallet *et al.*, 1990). There are two isoforms of the human nm23 gene, nm23-H1 and nm23-H2 (Stahl *et al.*, 1991). nm23-H1 and nm23-H2 have been shown to be identical respectively, to NDP kinases A and B from human erythrocytes (Gilles *et al.*, 1991).

The reduced expression of nm23 mRNA and protein is associated with increased metastatic ability of human breast carcinomas (Bevilacqua *et al.*, 1989; Hennessy *et al.*, 1991; Hirayama *et al.*, 1991; Royds *et al.*, 1993). When the nm23 cDNA is transfected into a metastatic murine melanoma cell line expressing low levels of this particular gene, transfected clones expressing the exogeneously induced nm23 gene show significantly reduced *in vivo* metastatic potential (Leone *et al.*, 1991). However, studies on colon carcinoma or neuroblastoma indicate increased nm23 gene expression to be associated with advanced stages of the disease (Hailat *et al.*, 1991; Leone *et al.*, 1993; Myeroff and Markowitz, 1993). Correlations between nm23 expression and metastatic potential would thus appear to depend on specific cancers.

Endometrial carcinoma is the most prevalent neoplasm of the female pelvis and has been detected in patients under the age of 45 (Jeffery *et al.*, 1987). There is no report on the expression of nm23 in endometrial carcinoma. Thus, in this study, examination was made of the expression of nm23-H1 and nm23-H2 in endometrial carcinomas by immunohistochemical and immunoblotting methods to clarify the association of nm23 expression with tumour metastatic potential, degree of differentiation, clinical stage, tumour size, myometrial invasion, oestrogen receptor (ER), progesterone receptor (PR) and menopause.

### Materials and methods

### *Tissue samples*

Thirty primary uterine malignant tumour specimens were surgically obtained from the pathology files of Kitasato University Hospital between 1992 and 1993. Assessment of clinical stage and the degree of differentiation was conducted according to the new classification of the International Federation of Gynecology and Obstetrics (FIGO, 1989). There were 15 well-differentiated (G1), five moderately differentiated (G2) and eight poorly differentiated adenocarcinomas (G3) and two leiomyosarcomas of the uterine corpus. Patient age ranged from 36 to 80 years, the median being 57 years. ER and PR in tumour tissue freshly obtained at surgery were measured by the radioreceptor assay system of Kitasato Biochemical Laboratory, Kanagawa, Japan. A tissue sample (0.5 $cm^3$) was also obtained and processed by AMeX, as described below.

### *AMeX procedure*

Sato *et al.* (1986, 1992) devised the AMeX (acetone, methylbenzoate, xylene) procedure for tissue processing and paraffin embedding before haematoxylin–eosin staining, immunostaining and immunoblotting. Morphological preservation of sections prepared by this method is consistently better than that of frozen sections and similar to that of routinely formalin-fixed paraffin sections. Proteins extracted from AMeX-processed tissue can be applied to immunoblotting. The technique is very simple for general clinical laboratory use, and paraffin blocks fixed by this method can be stored at 4°C for future study. Immunohistochemistry and immunoblotting were performed on the specimens obtained from the same block.

### *Immunohistochemistry*

Two mouse monoclonal antibodies (MAb), H1-229 (MAb for nm23-H1) and H2-439 (MAb for nm23-H2), were kindly provided by Dr Shiku, Department of Oncology, Nagasaki University, Nagasaki, Japan (Urano *et al.*, 1993). Paraffin-embedded samples were sectioned at 4 μm and deparaffinised with Histo-Clear (National Diagnostics, Atlanta, GA, USA). They were then immediately immersed in 4% paraformaldehyde for 5 min and washed with phosphate-buffered saline (PBS 0.0075 M pH 7.4). Immunohistochemical staining was carried out by the avidin–biotin–peroxidase complex (ABC) method. In brief, the sections were incubated with 2% normal swine serum and then with H1-229 (2 $\mu g\ ml^{-1}$) or H2-439 (4 $\mu g\ ml^{-1}$) overnight at room temperature, rinsed with PBS and incubated with biotinylated anti-mouse IgG (200-fold diluted, Vector, Burlingame, CA, USA) for 30 min. This was followed by reaction in 0.3% hydrogen peroxide in methanol for 30 min and then ABC and diaminobenzidine

Correspondence: J Watanabe
Received 2 May 1995; revised 28 June 1995; accepted 13 July 1995

reactions. Nuclear counterstaining was done using Mayer's haematoxylin solution. As negative controls, sections were stained by replacing the primary antibody with normal mouse serum at the same IgG concentration. Immunostaining intensity was categorized as strongly positive (++), positive (+) or negative (−) in comparison with background intensity.

*Immunoblotting*

Sections of cancer area were selectively cut from AMeX-processed tissue. They were deparaffinised with xylene and washed by centrifugation in acetone. All specimens were resuspended in Laemmli's sample buffer (Laemmli, 1970) by brief sonication and diluted to 4 mg protein $ml^{-1}$. Lysates of 10 μl (40 μg of protein) were heated at 100°C for 3 min and loaded onto a 15% SDS-polyacrylamide gel, which was then transferred to a nylon membrane (Immobilon-P, Millipore). The membrane was incubated with Block Ace (Dainihon Seiyaku, Suita, Osaka, Japan) and then with H1-229 (2 μg $ml^{-1}$) or H2-439 (2 μg $m^{-1}$) for 1 h, rinsed with PBS containing 0.3% Tween (PBS-T). This was followed by incubation with peroxidase-labelled anti-mouse IgG (1000-fold diluted, Dako, Kyoto, Japan) for 1 h. A band was detected on exposed X-ray film by a Western blot chemiluminescence reagent (DuPont, Boston, MA, USA). Densitometric scanning was performed with a CS 9000 densitometer (Shimazu, Kyoto, Japan) at 550 nm. The data were normalised as relative rates based on nm23 gene product content in the lysate (1 μg of protein) of the mouse myeloma cell line NS-1 transfected with either the nm23-H1 gene (NS-H1-9) or nm23-H2 gene (NS-H2-1) (Urano *et al.*, 1993) in the same membrane. NS-H1-9 and NS-H2-1 were also provided by Dr Shiku, Department of Oncology, Nagasaki University. Relative immunoblotting values were classed as 0, −; 0–0.8, +; over 0.8, ++; and were compared with those for immunostaining.

Statistical analysis was conducted by the Mann–Whitney *U*-test. *P*-values less than 0.05 were considered significant.

### Results

*Immunohistochemical analysis of nm23-H1 and -H2 protein*

The cytoplasm of cancer cells was stained diffusely with H1-229 (nm23-H1) and H2-439 (nm23-H2). There was no different staining pattern between the central area and the leading invasive edge of the tumour. Endometrial stromal cells showed negligible staining (Figure 1). The relationship between tumour differentiation and expression of nm23-H1 and -H2 protein as determined by immunohistochemical staining is shown in Table I. The staining intensity of nm23-H1 and -H2 was significantly stronger in G1 than in G2 ($P<0.05$) and stronger in G1 than in G3. No correlation could be found between the staining intensity of G1 and G3, since there were two strongly positive cases in eight G3 tumours. Nor was there any association of the immunoreactivity of nm23-H1 and -H2 with any other clinicopathological parameter (data not shown).

Two uterine leiomyosarcomas stained positively with MAb nm23-H1 and -H2. Two myometrial and two uterine cervical tissue specimens failed to stain by either MAb. Negative control sections were not stained.

*Immunoblotting analysis of nm23-H1 and -H2 protein*

Examples of nm23-H1 and -H2 protein expression of NS-H1-9 and NS-H2-1 cell lysates as positive controls and five endometrial carcinomas are shown in Figure 2. MAb H1-229 and H2-439 detected specific bands of 20.5 kDa (nm23-H1) and 18 kDa (nm23-H2) protein respectively.

Though the detection of nm23 gene products by immunostaining was possible, immunoblotting analysis could not be

**Figure 1** Immunohistochemical staining strongly positive for (**a**) nm23-H1 and (**b**) nm23-H2 protein in the cytoplasm of well-differentiated endometrial adenocarcinoma. (**c**) Positive staining for nm23-H1 protein and (**d**) negative staining for nm23-H2 protein in poorly differentiated endometrial adenocarcinoma. (ABC method, counterstained with haematoxylin, bar = 50 μm.)

**Table I** Immunohistochemical results for nm23-H1 and nm23-H2 gene products in relation to histological differentiation

| | | *Intensity of immunostaining* | | | | | | | |
|---|---|---|---|---|---|---|---|---|---|
| | | *nm23-H1* | | | | *nm23-H2* | | | |
| *Histology* | *No. of cases* | − | + | ++ | P-*value* | − | + | ++ | P-*value* |
| G1 | 15 | 0 | 6 | 9 | <0.05* (G1 vs G2) | 0 | 5 | 10 | <0.05* (G1 vs G2) |
| G2 | 5 | 1 | 4 | 0 | | 0 | 5 | 0 | |
| G3 | 8 | 1 | 5 | 2 | | 1 | 5 | 2 | |
| Leiomyosarcoma | 2 | 0 | 2 | 0 | | 0 | 2 | 0 | |
| Myometrium | 2 | 2 | 0 | 0 | | 2 | 0 | 0 | |
| Uterine cervix | 2 | 2 | 0 | 0 | | 2 | 0 | 0 | |

G1, well-differentiated adenocarcinoma; G2, moderately differentiated adenocarcinoma; G3, poorly differentiated adenocarcinoma; −, negative; +, positive; ++, strongly positive; *$P<0.05$, significant (Mann–Whitney $U$-test).

**Figure 2** Identification by immunoblotting analysis of (**a**) 20.5 kDa protein (nm23-H1) in lane H1 corresponding to the NS-H1-9 myeloma cell line transfected with nm23-H1 gene and five endometrial carcinomas (lanes 1–5) using MAb H1-229 and (**b**) 18 kDa protein (nm23-H2) in lane H2 corresponding to the NS-H2-1 myeloma cell line transfected with nm23-H2 gene and five endometrial carcinomas (lanes 1–5), using MAb H2-439. Values of densitometric analysis were normalised based on those in lanes H1 and H2.

conducted in two cases of G1, in one case because more than half the tissue had almost completely degenerated and in the other case because there was only a small portion of cancer area in the tissue. The relation between the expression of nm23-H1 and -H2 protein as determined by immunoblotting and clinicopathological data is shown in Table II.

Mean relative values of tumour nm23-H1 and -H2 protein in comparison with the positive controls, i.e. NS-H1-9 and NS-H2-1 cell lysates, were $0.7 \pm 0.4$ and $0.7 \pm 0.5$ in G1, $0.2 \pm 0.2$ and $0.2 \pm 0.2$ in G2 and $0.6 \pm 0.4$ and $0.5 \pm 0.4$ in G3. The values for nm23-H1 and -H2 were significantly higher in G1 than in G2 ($P<0.05$). No correlation was apparent between the values for nm23-H1 and -H2 in G1 and in G3 since there were two out of eight G3 tumour cases with high values of these parameters. The values for nm23-H1 and -H2 in two leiomyosarcomas were 0.1 and 1.2, and 0.1 and 0.1 respectively. Two myometrial and two uterine cervical tissue specimens showed no expression of nm23-H1 or -H2 protein.

*Comparison of nm23-H1 and -H2 protein expression determined by immunohistochemical and immunoblotting analysis*

Agreement rates of data obtained by immunohistochemical and immunoblotting analysis for nm23-H1 and -H2 protein expression were 69% (22/32) and 66% (21/32) respectively. Rates were higher by immunohistochemical analysis than by immunoblotting analysis were in 19% (6/32) and 25% (8/32) respectively. Rates were lower by immunohistochemical analysis than by immunoblotting analysis in 12% (4/32) and 9% (3/32) respectively.

*Correlation of nm23-H1 and -H2 protein expression based on immunoblotting and clinicopathological data*

The expression of nm23-H1 and -H2 showed no relation to stage, metastasis, tumour size, myometrial invasion, ER, PR or menopause in endometrical carcinoma by immunoblotting analysis (Table II).

### Discussion

In this study, assessment was made of the expression of nm23-H1 and nm23-H2 protein in endometrial carcinomas processed by the AMeX method (Sato *et al.*, 1986, 1992), in conjunction with immunohistochemical staining and immunoblotting. Agreement rates of the estimation of nm23-H1 and -H2 protein expression by immunostaining and immunoblotting were 69% and 66%. In disagreement cases, immunohistochemical analysis tended to be estimated as higher than immunoblotting, possibly owing to subjective estimation in immunostaining. It thus follows that it is more accurate to determine the localisation of nm23 protein expression by immunostaining and nm23 protein levels by immunoblotting.

No correlation could be detected between the expression of nm23-H1 and -H2 protein and putative prognostic factors such as stage, metastatic potential, tumour size, myometrial invasion, ER, PR and menopause. The reduced expression of nm23 mRNA and protein is associated with increased metastatic potential in human breast (Bevilacqua *et al.*, 1989; Hennessy *et al.*, 1991; Hirayama *et al.*, 1991; Royds *et al.*, 1993), hepatocellular (Nakayama *et al.*, 1992), gastric carcinomas (Nakayama *et al.*, 1993) and malignant melanoma (Flørenes *et al.*, 1992). Increased nm23 expression has been shown to be associated with advanced stages of colon carcinoma (Myeroff and Markowitz, 1993) and neuroblastoma (Hailat *et al.*, 1991; Leone *et al.*, 1993). No relationship between nm23 protein expression and clinicopathological factors has been demonstrated in breast (Sastre-Garau *et al.*, 1992; Sawan *et al.*, 1994) and pulmonary carcinoma (Higashiyama *et al.*, 1992). nm23-$\beta$ (analogous to human nm23-H1) and nm23-$\alpha$ (analogous to human nm23-H2) expression differs according to the organ in rat (Kimura *et al.*, 1990; Shimada *et al.*, 1993). The transfection of nm23 cDNA into a high metastatic K-1735 TK murine melanoma cell line significantly reduces *in vivo* metastatic potential (Leone *et al.*, 1991), but heterogeneity in nm23 expression among K-1735 clones and hybrids produced by the fusion of non-metastatic and metastatic K-1735 is not correlated with metastatic capability (Radinsky *et al.*, 1992). It would thus appear that

**Table II** Correlation between nm23-H1 and nm23-H2 protein expression by immunoblotting and clinicopathological parameters in patients with endometrial carcinoma

| *Clinicopathological parameters* | *No. of cases* | *nm23-H1* *Mean value* ± *s.d.*[a] | P-*value* | *nm23-H2* *Mean value* ± *s.d.*[a] | P-*value* |
|---|---|---|---|---|---|
| Histological grade | | | | | |
| G1 | 13 | 0.7 ± 0.4 | $P<0.05$* (G1 vs G2) | 0.7 ± 0.5 | $P<0.05$* (G1 vs G2) |
| G2 | 5 | 0.2 ± 0.2 | | 0.2 ± 0.2 | |
| G3 | 8 | 0.6 ± 0.4 | | 0.5 ± 0.4 | |
| Stage | | | | | |
| I A, B, C | 13 | 0.6 ± 0.5 | | 0.5 ± 0.5 | |
| II A, B | 4 | 0.3 ± 0.1 | NS | 0.4 ± 0.4 | NS |
| III A, B, C and IV A, B | 9 | 0.7 ± 0.4 | | 0.6 ± 0.5 | |
| Metastasis | | | | | |
| + | 7 | 0.6 ± 0.4 | NS | 0.5 ± 0.5 | NS |
| − | 19 | 0.5 ± 0.4 | | 0.5 ± 0.4 | |
| Tumour size | | | | | |
| ⩽ 5 cm | 12 | 0.6 ± 0.5 | NS | 0.5 ± 0.5 | NS |
| > 5 cm | 14 | 0.5 ± 0.3 | | 0.5 ± 0.4 | |
| Myometrial invasion | | | | | |
| − | 4 | 0.6 ± 0.5 | | 0.6 ± 0.6 | |
| ⩽ 1/2 | 12 | 0.6 ± 0.5 | NS | 0.5 ± 0.5 | NS |
| > 1/2 | 10 | 0.6 ± 0.4 | | 0.6 ± 0.4 | |
| ER | | | | | |
| + | 15 | 0.5 ± 0.4 | NS | 0.5 ± 0.4 | NS |
| − | 6 | 0.7 ± 0.4 | | 0.6 ± 0.5 | |
| PR | | | | | |
| + | 13 | 0.6 ± 0.5 | NS | 0.5 ± 0.5 | NS |
| − | 8 | 0.5 ± 0.3 | | 0.4 ± 0.4 | |
| Menopause | | | | | |
| Pre | 7 | 0.5 ± 0.4 | NS | 0.5 ± 0.5 | NS |
| Post | 19 | 0.6 ± 0.4 | | 0.5 ± 0.4 | |

[a]Value, relative value of tumour nm23-H1 and nm23-H2 protein based on positive controls, i.e. NS-H1-9 and NS-H2-1 cell lines. *$P<0.05$, significant (Mann–Whitney *U*-test); NS, not significant. G1, well-differentiated adenocarcinoma; G2, moderately differentiated adenocarcinoma; G3, poorly differentiated adenocarcinoma; Stage, new FIGO classification. ER, oestrogen receptor; PR, progesterone receptor; +, positive; −, negative; pre, premenopause; post, post-menopause.

any metastatic enhancer/suppressor function of nm23 may be tumour specific and/or the mechanism for nm23 expression may differ according to biochemical conditions.

The intensity of immunostaining of nm23-H1 and -H2 was significantly stronger in G1 than in G2 ($P<0.05$). nm23-H1 and -H2 protein were present significantly ($P<0.05$) more in G1 than in G2 according to immunoblotting analysis. Strong and weak intensity in G3 was apparent by immunostaining. The high content of nm23-H1 and -H2 protein would thus appear associated with the high degree of differentiation of endometrial carcinomas, while there may be two types of poorly differentiated endometrial adenocarcinomas. Confirmation of this will require study on a greater number of cases. nm23 mRNA and protein are expressed more in well-differentiated tumours of human breast cancer (Hennessy *et al.*, 1991; Royds *et al.*, 1993). Intense immunostaining of nm23-H1 in human prostate cancer has been detected more often in poorly differentiated than moderately differentiated types (Igawa *et al.*, 1994). The tissue- and developmental phase-specific expression of nm23 gene (*awd* gene) in *Drosophila* has been observed (Timmons *et al.*, 1993). The expression of nm23 may depend on primary sites and their differentiation.

nm23-H2 protein has been identified as the human c-*myc* transcription factor PuF, thus showing a relationship between nm23-H2 and c-*myc* oncogene expression and suggesting nm23-H2 protein to be essential to transcriptional activation of the c-*myc* gene (Postel *et al.*, 1993). The inverse relation between c-*myc* expression and cell differentiation is well documented (Spencer and Groudine, 1991) and nm23 has been shown to inhibit the differentiation of mouse myeloid leukaemia cells (Okabe-Kado *et al.*, 1992). The elevated expression of nm23 is associated with N-*myc* amplification in advanced stages of human neuroblastomas (Hailat *et al.*, 1991; Leone *et al.*, 1993). The mechanism for nm23 expression in well-differentiated tumours is still unknown. Poorly differentiated endometrial carcinomas highly expressing nm23-H2 may be independent of oestrogens and associated with c-*myc* overexpression.

In future study, clarification should be made of qualitative and quantitative relations between nm23-H2 and c-*myc* expression in endometrial carcinomas. It should also be determined whether nm23-H1 and -H2 have specific roles in tumour differentiation and if there are actually two types of endometrial carcinomas which express nm23 protein to different degrees.

**Acknowledgements**
This work was supported in part by Grants-in-Aid 05454455 and 06770134 from the Ministry of Education, Science and Culture, Japan. The authors are grateful to Dr Shiku for the gifts of H1-229, H2-439, NS-H1-9 and NH-H2-1 and to members of the Pathology Division of Kitasato University Hospital for preparing AMeX samples and to Ms B Sato for technical advice.

**References**

BEVILACQUA G, SOBEL ME, LIOTTA LA AND STEEG PS. (1989). Association of low nm23 RNA levels in human primary infiltrating ductal breast carcinomas with lymph node involvement and other histopathological indicators of high metastatic potential. *Cancer Res.*, **49**, 5185–5190.

FIGO (1989). FIGO news. *Int. J. Gynecol. Obstet.*, **28**, 189–193.

FLØRENES VA, AAMDAL S, MYKLEBOST O, MAELANDSMO GM, BRULAND ØS AND FODSTAD Ø. (1992). Levels of nm23 messenger RNA in metastatic malignant melanomas: inverse correlation to disease progression. *Cancer Res.*, **52**, 6088–6091.

GILLES AM, PRESECAN E, VONICA A AND LASCU I. (1991). Nucleoside diphosphate kinase from human erythrocytes. *J. Biol. Chem.*, **266**, 8784–8789.

HAILAT N, KEIM DR, MELHEM RF, ZHU XX, ECKERSKORN C, BRODEUR GM, REYNOLDS CP, SEEGER RC, LOTTSPEICH F, STRAHLER JR AND HANASH SM. (1991). High levels of p19/nm23 protein in neuroblastoma are associated with advanced stage disease and with N-*myc* gene amplification. *J. Clin. Invest.*, **88**, 341–345.

HENNESSY C, HENRY JA, MAY FEB, WESTLEY BR, ANGUS B AND LENNARD TWJ. (1991). Expression of the antimetastatic gene nm23 in human breast cancer: an association with good prognosis. *J. Natl Cancer Inst.*, **83**, 281–285.

HIGASHIYAMA M, DOI O, YOKOUCHI H, KODAMA K, NAKAMORI S, TATEISHI R AND KIMURA N. (1992). Immunohistochemical analysis of nm23 gene product/NDP kinase expression in pulmonary adenocarinoma: lack of prognostic value. *Br. J. Cancer*, **66**, 533–536.

HIRAYAMA R, SAWAI S, TAKAGI Y, MISHIMA Y, KIMURA N, SHIMADA N, ESAKI Y, KURASHIMA C, UTUYAMA M AND HIROKAWA K. (1991). Positive relationship between expression of anti-metastatic factor (nm23 gene product or nucleoside diphosphate kinase) and good prognosis in human breast cancer. *J. Natl Cancer Inst.*, **83**, 1249–1250.

IGAWA M, RUKSTALIS DB, TANABE T AND CHODAK GW. (1994). High levels of nm23 expression are related to cell proliferation in human prostate cancer. *Cancer Res.*, **54**, 1313–1318.

JEFFERY JD, TAYLOR R, ROBERTSON DI AND STUART GCE. (1987). Endometrial carcinoma occurring in patients under the age of 45 years. *Am. J. Obstet. Gynecol.*, **156**, 366–370.

KIMURA N, SHIMADA N, NOMURA K AND WATANABE K. (1990). Isolation and characterization of a cDNA clone encoding rat nucleoside disphosphate kinase. *J. Biol. Chem.*, **265**, 15744–15749.

LAEMMLI UK. (1970). Cleavage of structural proteins during the assembly of the head of bacteriophage T4. *Nature*, **227**, 680–685.

LEONE A, FLATOW U, KING CR, SANDEEN MA, MARGULIES IMK, LIOTTA LA AND STEEG PS. (1991). Reduced tumor incidence, metastatic potential, and cytokine responsiveness of nm23-transfected melanoma cells. *Cell*, **65**, 25–35.

LEONE A, SEEGER RC, HONG CM, HU YY, ARBOLEDA MJ, BRODEUR GM, STRAM D, SLAMON DJ AND STEEG PS. (1993). Evidence for nm23 RNA overexpression, DNA amplification and mutation in aggressive childhood neuroblastoma. *Oncogene*, **8**, 855–865.

MYEROFF LL AND MARKOWITZ SD. (1993). Increased nm23-H1 and nm23-H2 messenger RNA expression and absence of mutations in colon carcinomas of low and high metastatic potential. *J Natl Cancer Inst.*, **85**, 147–152.

NAKAYAMA H, YASUI W, YOKOZAKI H AND TAHARA E. (1993). Reduced expression of nm23 is associated with metastasis of human gastric carcinomas. *Jpn. J. Cancer Res.*, **84**, 184–190.

NAKAYAMA T, OHTSURU A, NAKAO K, SHIMA M, NAKATA K, WATANABE K, ISHII N, KIMURA N AND NAGATAKI S. (1992). Expression in human hepatocellular carcinoma of nucleoside diphosphate kinase, a homologue of the nm23 gene product. *J. Natl Cancer Inst.*, **84**, 1349–1354.

OKABE-KADO J, KASUKABE T, HONMA Y, HAYASHI M, HENZEL WJ AND HOZUMI M. (1992). Identity of a differentiation inhibiting factor for mouse myeloid leukemia cells with nm23/nucleoside diphosphate kinase. *Biochem. Biophys. Res. Commun.*, **182**, 987–994.

POSTEL EH, BERBERICH SJ, FLINT SJ AND FERRONE CA. (1993). Human c-*myc* transcription factor PuF identified as nm23-H2 nucleoside disphosphate kinase, a candidate suppressor of tumor metastasis. *Science*, **261**, 478–480.

RADINSKY R, WEISBERG HZ, STAROSELSKY AN AND FIDLER IJ. (1992). Expression level of the nm23 gene in clonal populations of metastatic murine and human neoplasms. *Cancer Res.*, **52**, 5808–5814.

ROSENGARD AM, KRUTZSCH HC, SHEARN A, BIGGS JR, BARKER E, MARGULIES IMK, KING CR, LIOTTA LA AND STEEG PS. (1989). Reduced nm23/awd protein in tumour metastasis and aberrant *Drosophila* development. *Nature*, **342**, 177–180.

ROYDS JA, STEPHENSON TJ, REES RC, SHORTHOUSE AJ AND SILCOCKS PB. (1993). Nm23 protein expression in ductal in situ and invasive human breast carcinoma. *J. Natl Cancer Inst.*, **85**, 727–731.

SASTRE-GARAU X, LACOMBE ML, JOUVE M, VÉRON M AND MAGDELÉNAT H. (1992). Nucleoside diphosphate kinase/nm23 expression in breast cancer: Lack of correlation with lymph-node metastasis. *Int. J. Cancer*, **50**, 533–538.

SATO Y, MUKAI K, WATANABE S, GOTO M AND SHIMOSATO Y. (1986). The AMeX method: a simplified technique of tissue processing and paraffin embedding with improved preservation of antigens for immunostaining. *Am. J. Pathol.*, **125**, 431–435.

SATO Y, MUKAI K, FURUYA S, KAMEYA T AND HIROHASHI S. (1992). The AMeX method: a multipurpose tissue-processing and paraffin-embedding method, extraction of protein and application to immunoblotting. *Am. J. Pathol.*, **140**, 775–779.

SAWAN A, LASCU I, VERON M, ANDERSON JJ, WRIGHT C, HORNE CHW AND ANGUS B. (1994). NDP-K/nm23 expression in human breast cancer in relation to relapse, survival, and other prognostic factors: an immunohistochemical study. *J. Pathol.*, **172**, 27–34.

SHIMADA N, ISHIKAWA N, MUNAKATA Y, TODA T, WATANABE K AND KIMURA N. (1993). A second form ($\beta$ isoform) of nucleoside diphosphate kinase from rat: isolation and characterization of complementary and genomic DNA expression. *J. Biol. Chem.*, **268**, 2583–2589.

SPENCER CA AND GROUDINE M. (1991). Control of c-*myc* regulation in normal and neoplastic cells. *Adv. Cancer Res.*, **56**, 1–48.

STAHL JA, LEONE A, ROSENGARD AM, PORTER L, KING CR AND STEEG PS. (1991). Identification of a second human nm23 gene, nm23-H2. *Cancer Res.*, **51**, 445–449.

STEEG PS, BEVILACQUA G, KOPPER L, THORGEIRSSON UP, TALMADGE JE, LIOTTA LA AND SOBEL ME. (1988). Evidence for a novel gene associated with low tumor metastatic potential. *J Natl Cancer Inst.*, **80**, 200–204.

TIMMONS L, HERSPERGER E, WOODHOUSE E, XU J, LIU LZ AND SHEARN A. (1993). The expression of the *Drosophila awd* gene during normal development and in neoplastic brain tumors caused by *lgl* mutasions. *Dev. Biol.*, **158**, 364–379.

URANO T, FURUKAWA K AND SHIKU H. (1993). Expression of nm23/NDP kinase proteins on the cell surface. *Oncogene*, **8**, 1371–1376.

WALLET V, MUTZEL R, TROLL H, BARZU O, WURSTER B, VERON M AND LACOMBE ML. (1990). *Dictyostelium* nucleoside diphosphate kinase highly homologous to mm23 and awd proteins involved in mammalian tumor metastasis and *Drosophila* development. *J. Natl Cancer Inst.*, **82**, 1199–1202.